# Atopic Dermatitis–like mouse model using early inoculation of patient-derived *S. aureus* together with MC903

**DOI:** 10.1016/j.xjidi.2025.100436

**Published:** 2025-11-21

**Authors:** Aaroh Joshi, Altan Cornu, Josefa Luxner, Gernot Zarfel, Camille Braun, Jean-Francois Nicolas, Richard L. Gallo, Marc Vocanson, Peter Wolf, Vijaykumar Patra

**Affiliations:** 1 Department of Dermatology and Venereology, Medical University of Graz, Graz, Austria; 2 Department of Dermatology, University of California San Diego, La Jolla, California, USA; 3 CIRI (Centre International de Recherche en Infectiologie), INSERM, U1111, Univ Lyon, Université de Lyon 1, Ecole Normale Supérieure de Lyon, CNRS, UMR 5308, Lyon, France; 4 Institute for Hygiene, Microbiology and Environmental Medicine, Medical University Graz, Graz, Austria; 5 Allergy and Clinical Immunology Department, Lyon Sud University Hospital, Pierre-Bénite, Lyon, France

**Keywords:** Atopic dermatitis, *S. aureus*, *S. epidermidis*, Strain-level variation, Mouse model, Type 2 immunity, Microbiome–host interactions, *S. aureus* genome, MC903

## Abstract

*Staphylococcus aureus* (*S. aureus*) worsens atopic dermatitis (AD), but how individual strains differ in pathogenicity remains unclear. Mouse models that mimic AD and allow direct manipulation of *S. aureus* in early stages of disease are limited. Moreover, these models rarely incorporate clinical *S. aureus* strains isolated from patients with AD. In this study, we investigated the inflammatory potential of clinical *S. aureus* and *S. epidermidis* isolates from patients with AD in a mouse model. Clinical *S. aureus* strains showed significant variability in their ability to elicit inflammation. The inflammation was associated with differences in virulence factor expression and, to a lesser extent, with genomic variation. In contrast, *S. epidermidis* strains (taken from the same lesional skin sites of patients) induced only mild but consistent inflammation, with less variability at the strain level. Next, we examined the impact of a pathogenic clinical *S. aureus* strains in the presence of an MC903-induced type 2 immune environment. Under these conditions, *S. aureus* enhanced colonization; increased inflammation; and promoted type 1, type 2, and type 17/22 immune responses. These responses were less evident with either treatment alone. Our findings suggest that clinical *S. aureus* strains from patients with AD differ in their capacity to modulate skin inflammation, particularly within a type 2–skewed environment. These results highlight the potential value of incorporating clinically relevant *S. aureus* isolates into early-stage in vivo models to better understand AD immunopathology and to inform microbiome-targeted therapeutic strategies.

## Graphical abstract

**Figure:**
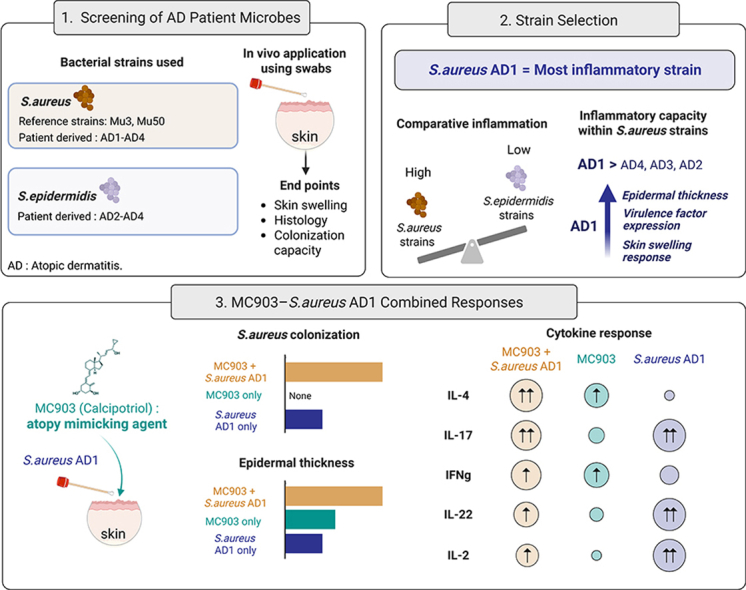

## Introduction

Atopic dermatitis (AD) is a chronic inflammatory skin condition with a lifetime prevalence of up to 20% (Deckers et al, 2012). AD manifests with diverse symptoms, including eczematous lesions, red and scaly patches, and intense pruritus (Weidinger et al, 2018). The pathogenesis of AD is multifactorial, involving 3 interconnected features: epidermal barrier dysfunction, a type 2–dominated immune response, and skin microbial dysbiosis (Beck et al, 2022; Brunner and Guttman-Yassky, 2019; Czarnowicki et al, 2019; Okamoto et al, 2025).

It has long been recognized that patients with AD are more frequently colonized by *Staphyloccocus aureus* (*S. aureus*) (Leyden et al, 1974). Whether *S. aureus* can initiate AD remains uncertain owing to conflicting findings (Kennedy et al, 2017; Meylan et al, 2017). It is suggested that in a subset of patients, *S. aureus* may act as a causal factor in disease onset (Williams and Gallo, 2017). Nonetheless, its role in AD progression is well-established. *S. aureus* is now recognized as a key contributor to AD pathogenesis, driving disease through the disruption of the skin barrier, induction of itch, and modulation of immune responses to a broad array of virulence factors (Braun et al, 2024; Byrd et al, 2017; Geoghegan et al, 2018; Obata et al, 2023; Lane Starr et al, 2024; Wang et al., 2024a).

As a result of these findings, *S. aureus* has emerged as a key therapeutic target in the management of AD. Advances have been made in the development of *S. aureus*–targeting therapies. Notable approaches include bacteriotherapy with commensal bacteria (Nakatsuji et al, 2021), antimicrobial agents such as niclosamide (Weiss et al, 2022), engineered endolysins (Eichenseher et al, 2022), inhibitors of the *S. aureus* agr quorum-sensing system (Williams et al, 2019), and the potential use of antimicrobial peptides or bacteriocins (Joshi et al, 2023).

*Staphylococcus epidermidis* (*S. epidermidis*) is another common skin commensal that is frequently found in the skin of patients with AD and was recently shown to play a pathogenic role in AD (Cau et al, 2021; Landemaine et al, 2023). Its immune-modulating effects are now recognized as strain dependent. For instance, strains belonging to the A20 clade have been shown to induce CD8+ T-cell responses and are less prevalent in AD skin than in healthy skin (Linehan et al, 2018). On the other hand, in vitro studies suggest an immunosuppressive role for certain *S. epidermidis* strains (Lai et al, 2009; Yi Pan et al, 2025). Notably, toll-like receptor 2 activation by *S. epidermidis–*derived lipoteichoic acid induces a distinct IL-10 immune signature, a response not observed with *S. aureus*–derived lipoteichoic acid (Volz et al, 2018). However, this immunosuppressive effect has not been demonstrated in in vivo mouse models or ex vivo skin tissue studies. These findings highlight the need for further research to clarify the strain-specific role of *S. epidermidis* in AD pathogenesis.

Despite their utility, current AD mouse models lack the sophistication needed to fully replicate human AD features and often overlook the role of *S. aureus* (Gilhar et al, 2021; Kim et al, 2019). It is known that individuals with AD who are colonized by *S. aureus* display unique phenotypic and endotypic characteristics (Simpson et al, 2018). Models utilizing the vitamin D analog calcipotriol (MC903), which induces thymic stromal lymphopoietin expression in keratinocytes, as well as epicutaneous sensitization with allergens such as house dust mite extract or ovalbumin combined with barrier disruption are commonly employed owing to their relative ease of use and reproducibility (Jin et al, 2009; Matsuoka et al, 2003; Moosbrugger-Martinz et al, 2017; Spergel et al, 1998). Spontaneous mouse mutants, such as Nc/Nga and flaky tail mice, also replicate aspects of human AD (Fallon et al, 2009; Matsuda et al, 1997; Scharschmidt et al, 2009). Certain AD-like models exist, where live *S. aureus* is introduced only after type 2 inflammation has been established after MC903 or ovalbumin treatment, on the basis of the assumption that *S. aureus* colonization is secondary to the development of type 2 inflammation (Leyva-Castillo et al, 2020). This approach does not examine the events involving *S. aureus* in the context of subclinical type 2 microenvironment, which simulates early disease conditions. In addition, the use of nonclinical isolates limits the translational relevance of these models (Paller et al, 2019).

To address these gaps, we developed a mouse model incorporating a clinical pathogenic isolate of *S. aureus* together with MC903. This synergistic model demonstrated clinical, histological, and cytokine profiles closely resembling those of patients with AD. This preclinical tool provides a platform for studying *S. aureus*’s role in AD pathogenesis, particularly during the early disease phases, and facilitates the testing of novel *S. aureus*–targeting therapeutic strategies.

## Results

### Clinical *S. aureus* strains from patients with AD trigger diverse and strain-specific inflammatory responses upon topical application in mice

**Figure 1:**
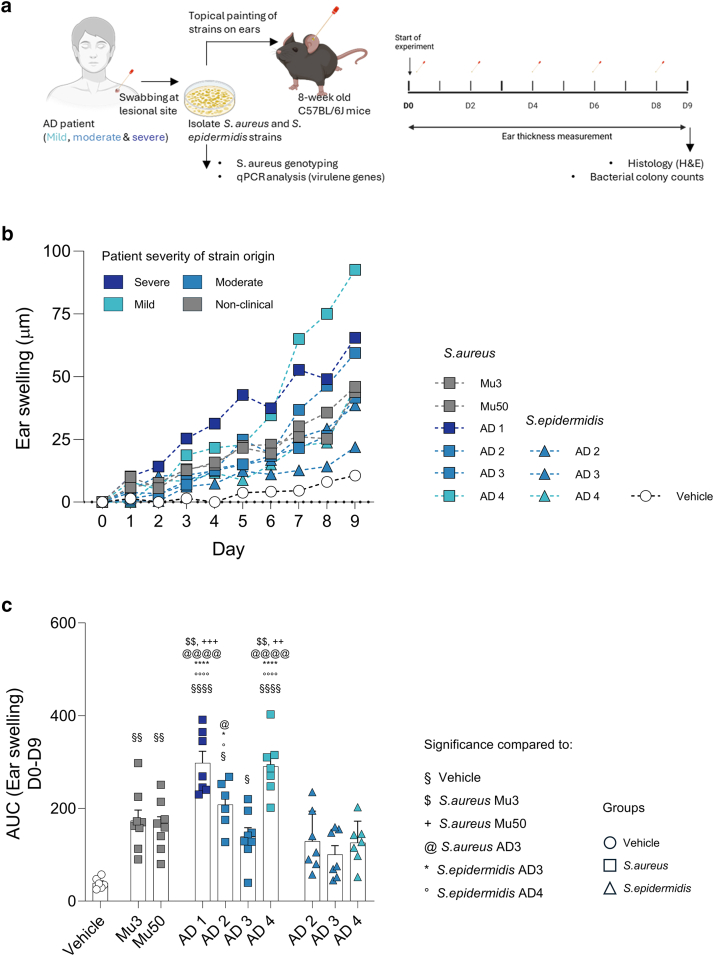
**Strain-dependent ear swelling responses of clinical isolates upon topical application.** (**a**) Experimental protocol: nonclinical *S. aureus* strains Mu3 and Mu50, AD patient–derived *S. aureus* and *S. epidermidis* strains (AD1–AD4), and TSB as a control vehicle were topically applied to the ears of C57BL/6 mice on days 0, 2, 4, 6, and 8. Clinical skin inflammation was assessed by measuring ear thickness. (**b**) Ear swelling over time: mean ear thickness (μm) from day 0 (denoted as D0) to day 9 (denoted as D9) for each treatment group (n = 6–8 mice per group). (**c**) Cumulative ear swelling: individual values and group mean ± SEM for the AUC of ear thickness measurements from D0 to D9 (n = 6–8 mice per group). Statistical analysis: 1-way ANOVA followed by Tukey’s multiple comparisons test. ∗*P* < .05, ∗∗*P* < .01, and ∗∗∗*P* < .001. Symbols indicate statistical significance compared with the following groups: § vehicle, $ *S. aureus* Mu3, + *S. aureus* Mu50, @ *S. aureus* AD3, ∗ *S. epidermidis* AD3, and ° *S. epidermidis* AD4. AUC, area under the curve; TSB, tryptic soy broth.

**Figure 2:**
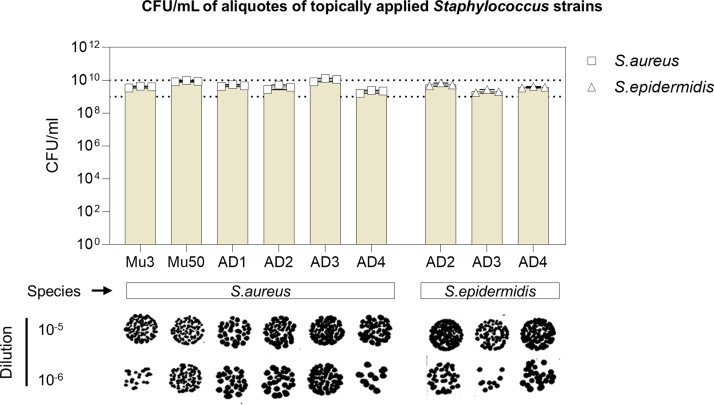
**Quantification of inoculum density of applied Staphylococcus strains.** CFU counts of frozen aliquots of *S. aureus* and *S. epidermidis* strains used for topical application in this study. Top: CFU concentrations (CFU/ml) determined by retrospective plating of inocula onto tryptic soy agar. Bars represent mean ± SEM of 3 independent measurements (n = 3), with individual data points overlaid (squares: *S. aureus*; triangles: *S. epidermidis*). CFU densities ranged from approximately 10^9^ to 10^10^ CFU/ml across all strains. Bottom: representative images of dilution plates (10^-5^ and 10^-6^ dilutions) illustrating colony morphology and density for each strain. CFU, colony-forming unit.

To investigate how clinical *S. aureus* and *S. epidermidis* strains influence inflammatory responses in the intact skin of mice, we isolated bacterial strains from the lesional skin of patients with AD with varying levels of severity (Table 1). In addition, we included 2 nonclinical *S. aureus* strains (Mu3 and Mu50), which are genetically similar to AD04.E17, a strain previously shown to induce AD-like immunological responses upon topical application in mice (Braun et al, 2024; Byrd et al, 2017). All bacterial suspensions were standardized to an optical density at 600 nm and applied topically to intact dorsal skin of mice every other day (Figure 1a). Although there was some strain-to-strain variation in colony-forming unit (CFU)/ml, all inocula consistently fell within the range of 10^9^–10^10^ CFU/ml (Figure 2).

**Table 1:** Clinical Characteristics of Patients with AD from Whom the *S. aureus* Strains Were Isolated

Patient Code	Sex	Age, y	Site of Strain Isolation	EASI	Severity Grade	IgE Total (0–1000 kU/l)	Family History of Atopic Diseases	Comorbidities
AD1	F	35	Inner elbow region	34	Severe	1547	AD (mother and sister)	No
AD2	F	52	Volar wrist	12.3	Moderate	3570	No	Factor –V Leiden sequence variation
AD3	F	25	Mid back	5.7	Moderate	18.4	No	No
AD4	F	30	Inner elbow region	3.3	Mild	760	No	Wasp/hornet venom allergy; severe bronchial asthma; migraine with aura; recurrent food anaphylaxis

Abbreviations: AD, atopic dermatitis; EASI, Eczema Area and Severity Index; F, female.

The table summarizes demographic and clinical data from 4 patients with AD (AD1–AD4) from whom *S. aureus* strains were isolated. Parameters include age, sex, site of strain isolation, EASI score, severity grade of AD, total serum IgE levels, family history of atopic diseases, and comorbidities.

Significant differences in ear swelling were observed between *S. aureus/S. epidermidis–*colonized and noncolonized mice after the application of these bacterial strains (Figure 1b and c). *S. aureus* strains induced a higher skin swelling than the *S. epidermidis* strains isolated from the same lesions. On day 5, ear swelling was highest with the *S. aureus* AD1 strain (*P* < .001), followed by *S. aureus* AD4 (*P* = .014). However, on days 8 and 9, ear swelling was greater with the *S. aureus* AD4 strain. When the total ear swelling was calculated as the area under the curve of recorded values, *S. aureus* AD1 and AD4 strains showed the highest capacity to trigger an immune response (measured as ear swelling), which was significant compared with the nonclinical strains (Mu3 and Mu50), *S. aureus* AD3, and all *S. epidermidis* strains. Among the *S. epidermidis* strains, no significant differences in ear swelling were observed.

**Figure 3:**
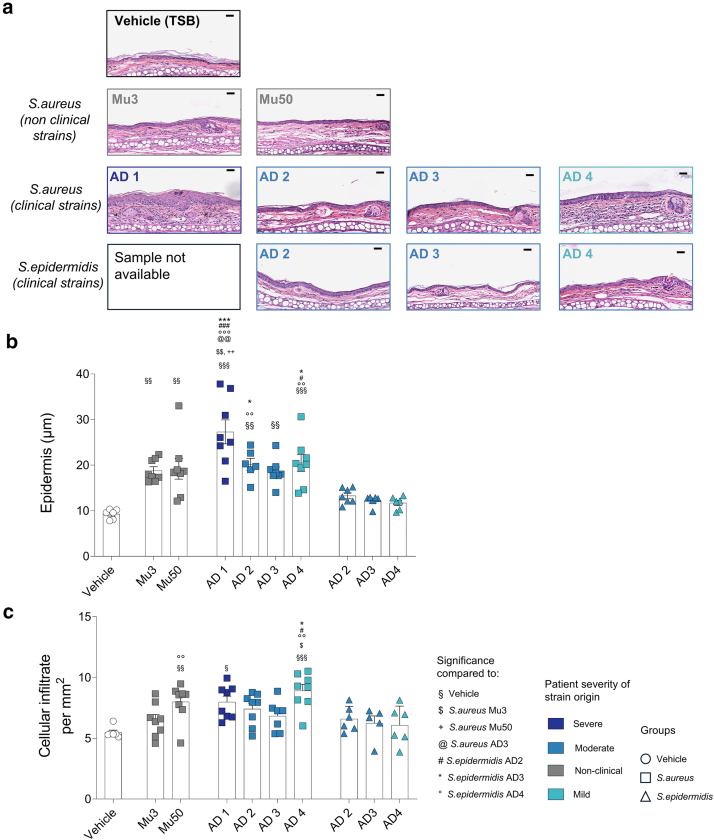
**Histological analysis of mouse ear skin after treatment with bacterial strains.** (**a**) Representative images: H&E-stained sections of mouse ear skin collected on day 9 after treatment with *S. aureus* (Mu3, Mu50, AD1–AD4), *S. epidermidis* (AD-derived strains), or TSB. Bar = 50 μm. (**b**) Epidermal thickness: mean epidermal thickness (μm) ± SEM from H&E-stained sections (n = 6–8 mice per group). (**c**) Dermal cellular infiltration: mean dermal cell infiltration ± SEM from the same histological images (n = 5–8 mice per group). Statistical analysis: Kruskal–Wallis test followed by Dunn’s multiple comparisons test. ∗*P* < .05, ∗∗*P* < .01, and ∗∗∗*P* < .001. Symbols indicate statistical significance compared with the following groups: § vehicle, $ *S. aureus* Mu3, + *S. aureus* Mu50, @ *S. aureus* AD3, ∗ *S. epidermidis* AD3, and ° *S. epidermidis* AD4. AD, atopic dermatitis; TSB, tryptic soy broth.

Histological analysis of mouse ear skin sections stained with H&E on day 9 demonstrated increased epidermal thickness in all *S. aureus*–treated groups compared with that in the vehicle control (Figure 3a and b). Among the *S. aureus* strains, AD1 induced the greatest epidermal thickness. Consistent with ear swelling measurements, *S. aureus*–treated mice exhibited greater epidermal thickening than those treated with *S. epidermidis* strains.

We next quantified dermal cellular infiltrate by normalizing cell counts to tissue area. H&E-stained sections were analyzed using QuPath, which identified cell nuclei on the basis of hematoxylin staining. The density of cellular infiltrate in *S. aureus*–treated mouse ear skin (Mu50, AD1, and AD4) was higher than that in the vehicle control (Figure 3c). In addition, cellular infiltration was greater in the *S. aureus* AD4–treated group than in the *S. epidermidis–*treated groups.

**Figure 4:**
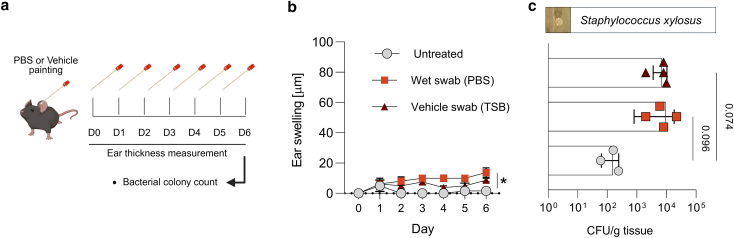
**Effect of vehicle control treatments on mouse ear skin.** (**a**) Experimental design. Mice received daily applications of either PBS-moistened swab (wet swab) or TSB-moistened swabs (vehicle swab) or were left untreated over 6 days. Ear thickness was measured daily, and bacterial load was quantified at day 6. (**b**) Ear swelling over time. Change in ear thickness (μm) over 6 days in untreated (open circles), PBS-swabbed (red squares), and TSB-swabbed (brown triangles) groups (n = 3–4 mice per group). Data are shown as mean ± SEM. Statistical analysis was performed using Kruskal–Wallis test followed by Dunn’s multiple comparisons. (**c**) Bacterial colonization. CFU per gram of ear tissue at day 6 in untreated, PBS-swabbed, and TSB-swabbed mice (n = 3–4 per group). Bars show mean ± SEM with individual values overlaid. *S xylosus* was identified as the predominant colonizer (representative colony morphology shown). No significant differences in bacterial load were detected between groups (Kruskal–Wallis with Dunn’s posthoc). CFU, colony-forming unit; D0, day 0; D1, day 1; D2, day 2; D3, day 3; D4, day 4; D5, day 5; D6, day 6; TSB, tryptic soy broth.

We observed that the vehicle itself induced very minimal inflammation. To determine whether this effect was due to the media or to handling, we conducted a follow-up control experiment in which mice were swabbed daily with either PBS or tryptic soy broth (TSB) (vehicle) or were left untreated (no swab) (Figure 4a). Both the PBS- and TSB-swabbed groups showed mild ear swelling compared with untreated controls, with no significant difference between them, suggesting that the minimal inflammation was primarily attributable to handling rather than the media composition (Figure 4b). Microbial plating revealed increased CFU counts in both swabbed groups. Colonies isolated from TSB-swabbed ears were identified using matrix-assisted laser desorption ionization–time of flight as *S xylosus*, a commensal bacterium commonly found on murine skin.

### Inflammatory responses are independent of differences in the colonization capacity of *S. aureus* strains

**Figure 5:**
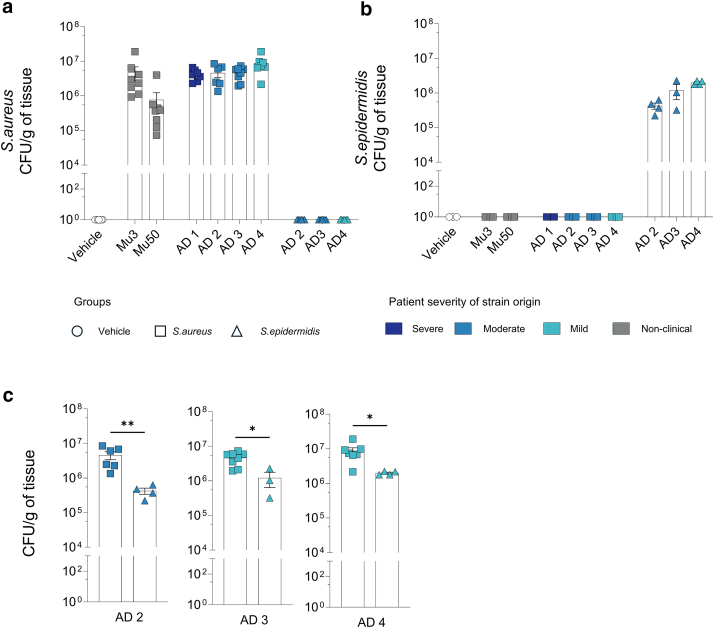
**Bacterial colonization of mouse ear skin after treatment with clinical isolates.** (**a**) *S. aureus* colonization: CFUs per gram of tissue recovered from mouse ears on day 9 after treatment with *S. aureus* strains or TSB. Data are shown as individual values with mean ± SEM (n = 3–8 mice per group). (**b**) *S. epidermidis* colonization: CFU per gram of tissue recovered from mouse ears on day 9 after treatment with *S. epidermidis* strains (AD derived) or vehicle. Data are shown as individual values with mean ± SEM (n = 3–8 mice per group). (**c**) Comparison of bacterial colonization between clinical isolates: *S. aureus* or *S. epidermidis* CFU per gram of tissue for AD2, AD3, and AD4 strains at day 9. Data are shown as individual values with mean ± SEM (n = 3–8 mice per group). Statistical analysis: Mann–Whitney test; ∗*P* < .05 and ∗∗*P* < .01. AD, atopic dermatitis; CFU, colony-forming unit; TSB, tryptic soy broth.

**Figure 6:**
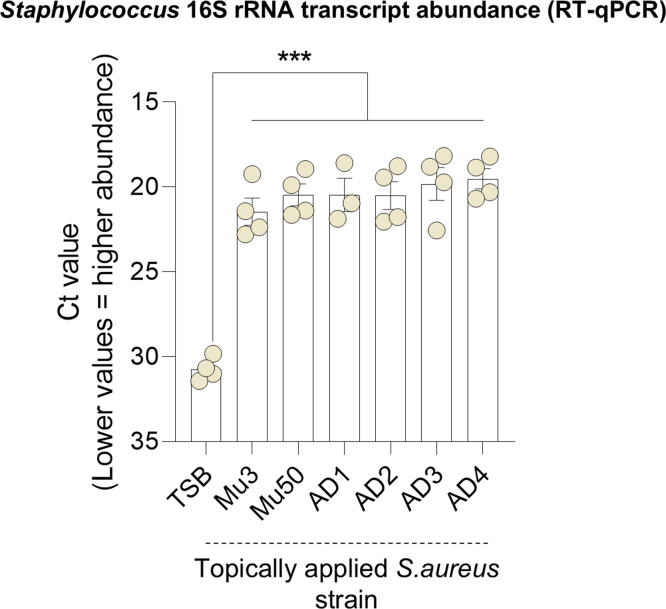
***S. aureus* colonization levels on mouse ear skin.** Ear tissue from mice colonized with different *S. aureus* strains was analyzed by RT-qPCR targeting *Staphylococcus* 16S rRNA to estimate bacterial burden. Lower cycle threshold (Ct) values indicate higher bacterial abundance. All colonized groups showed significantly greater *S. aureus* abundance than the vehicle (TSB) (1-way ANOVA with Tukey’s posthoc test, ∗∗∗*P* < .001), with no significant differences detected among the *S. aureus* strains themselves. Bars represent mean ± SD of biological replicates (n = 3–4 per strain), with individual values overlaid. rRNA, ribosomal RNA; TSB, tryptic soy broth.

We investigated whether the observed inflammatory differences between bacterial strains were attributable to variations in their colonization capacities. To assess this, we quantified the CFUs per gram of tissue on day 9. As expected, no *S. aureus* colonies were detected in the skin treated with either the vehicle control or *S. epidermidis* strains (Figure 5a). In contrast, ear skin from mice treated with *S. aureus* strains exhibited colonization levels ranging from 10^5^ to 10^7^ CFU/g of tissue. Among the *S. aureus*–treated mice, there were no significant differences in colonization levels across strains, except for the nonclinical *S. aureus* Mu50 strain, which showed lower colonization. Consistent with these CFU-based findings, reverse transcription quantitative PCR quantification of *Staphylococcus* 16S ribosomal RNA transcript levels similarly revealed no significant differences in colonization among *S. aureus–*treated group (Figure 6). This suggests that colonization capacity does not directly correlate with the inflammatory potential within strains of *S. aureus* species.

In *S. aureus*–treated mice, as expected, no colonies of *S. epidermidis* were detected (Figure 5b). Within the *S. epidermidis*–treated groups, minor strain-specific differences in colonization were observed, with the *S. epidermidis* AD4 strain showing the slightly increased colonization levels. Direct comparison of matched AD strains from both species revealed that *S. aureus* colonized skin significantly more efficiently than *S. epidermidis* (Figure 5c).

### Complex interplay of virulence factor expression may underpin inflammatory diversity in *S. aureus s*trains

Because the inflammatory response was not associated with colonizing potential of the microbes, we hypothesized that other microbial components such as virulence factors may play a role and explain the strain-level differences observed in triggering an immune response. In fact, previous studies have established links between genomic differences, virulence factor expression patterns, and the status and severity of AD (Key et al, 2023; Saheb Kashaf et al, 2023). Therefore, we analyzed the genomic similarities and expression of virulence factors among the *S. aureus* strains.

**Figure 7:**
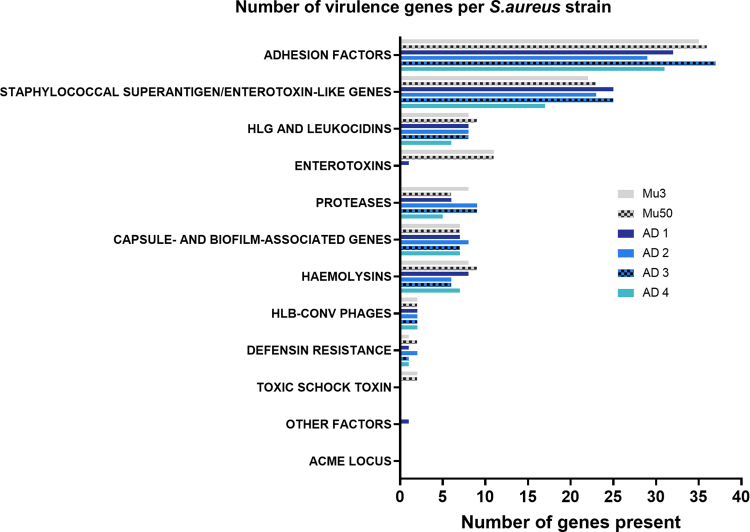
**Distribution of virulence genes across *S. aureus* strains.** Virulence gene content of 6 *S. aureus* strains (Mu3, Mu50, AD1, AD2, AD3, and AD4) was analyzed by microarray and grouped by functional categories. Bars represent the number of genes identified within each category per strain. Categories include adhesion factors, staphylococcal superantigen/enterotoxin-like genes, HLG and leukocidins, enterotoxins, proteases, capsule- and biofilm-associated genes, hemolysins, HLb-converting phages, defensin resistance, toxic shock toxin, ACME locus, and other factors. The plot illustrates variation in virulence gene repertoire between strains, with adhesion factors and enterotoxin-like genes showing the highest representation. ACME, arginine catabolic mobile element; HLG, hemolysin .

**Figure 8:**
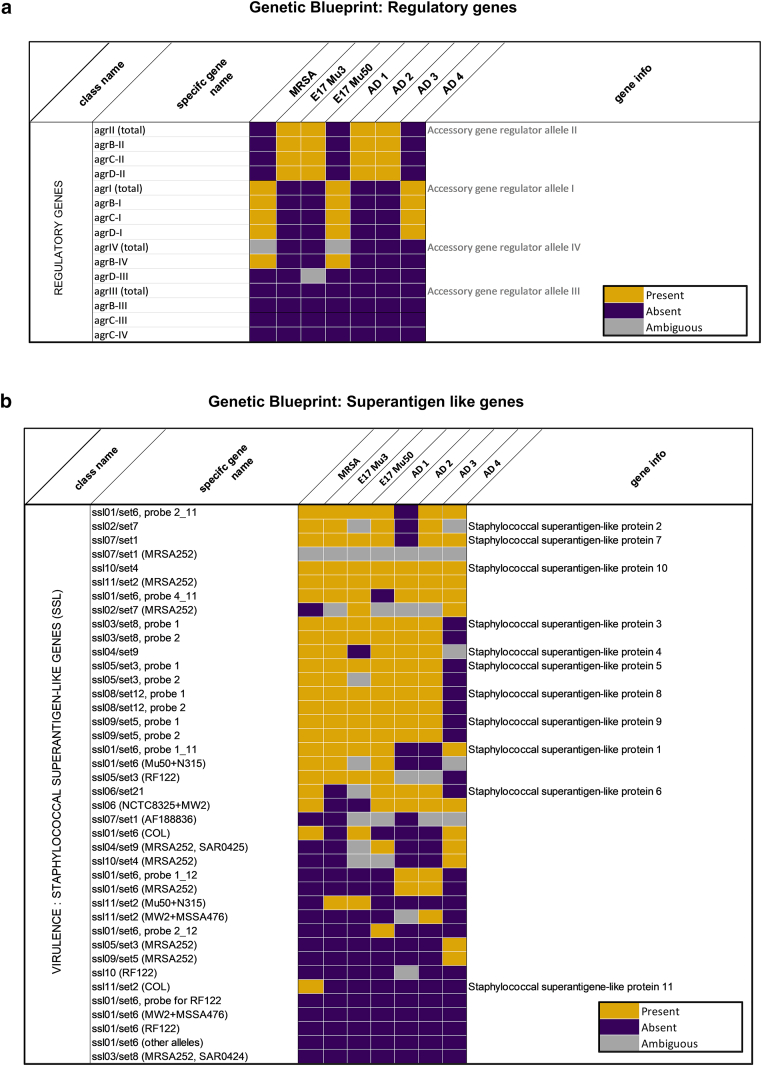

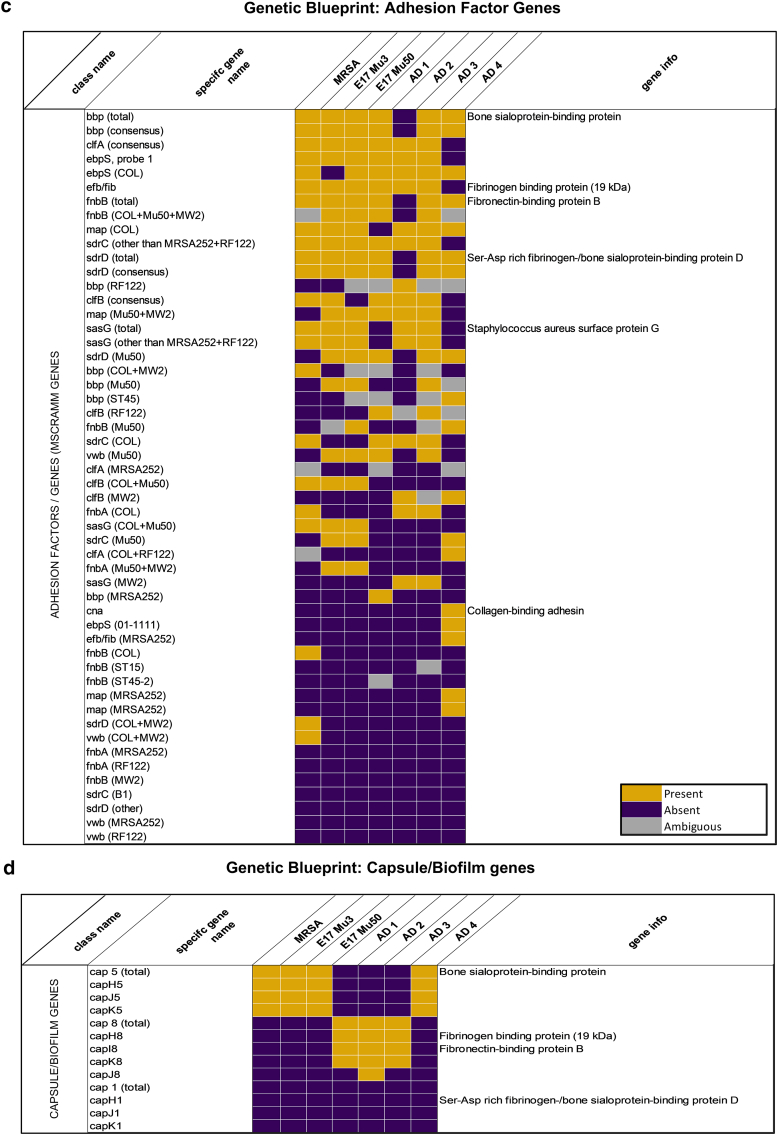

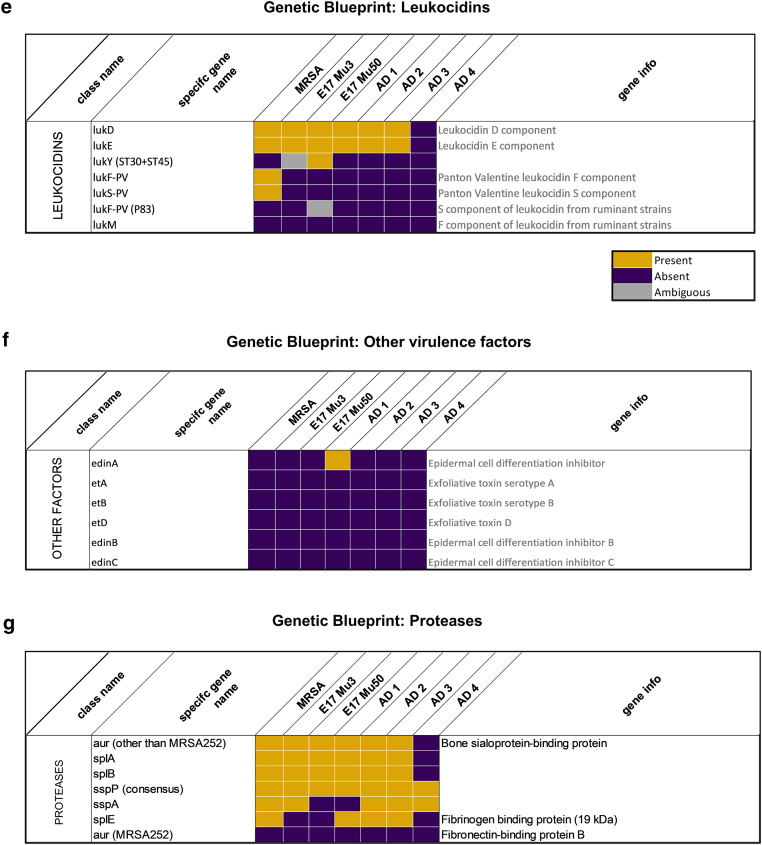
**Comparative genomic presence of virulence- and regulation-associated gene families in *S. aureus* Strains.** Heatmaps depict the distribution of gene families associated with (**a**) regulation, (**b**) superantigen-like proteins, (**c**) adhesion, (**d**) biofilm formation, (**e**) leukocidins, (**f**) unconventional virulence factors, and (**g**) proteases across 7 *S. aureus* strains (MRSA USA300, Mu3, Mu50, AD1, AD2, AD3, and AD 4). Each cell represents the status of a gene in a given strain: presence (yellow), absence (purple), or ambiguous (gray). MRSA, Methicillin-Resistant *Staphylococcus aureus*.

**Figure 9:**
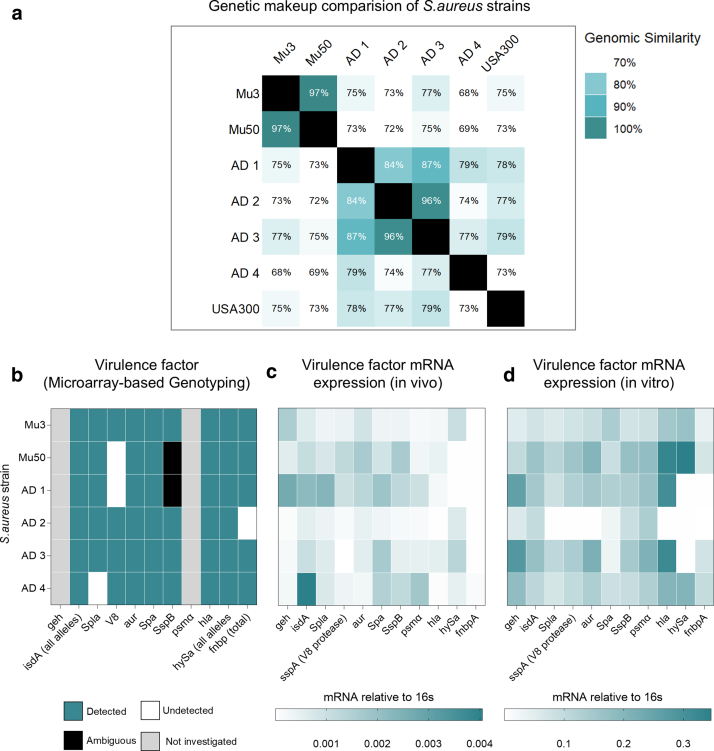
**Molecular characterization of *S. aureus* strains recovered from mouse ear skin.** (**a**) Genomic similarity of *S. aureus* strains based on microarray-based genotyping. Values indicate the percentage overlap of detected genes between strains, with shading corresponding to 70–100% similarity. (**b**) Virulence factor gene carriage determined by microarray profiling. Each square represents the presence (teal), absence (white), ambiguous detection (black), or not investigated (gray) status of selected virulence-associated genes in each strain. (**c**) In vivo virulence factor mRNA expression, quantified by RT-qPCR from infected mouse ear skin (biological replicates n = 3–4 per strain). Expression values were normalized to the 16S rRNA housekeeping gene and are shown as relative abundance (ΔCt values converted to relative expression). (**d**) In vitro virulence factor mRNA expression, determined by RT-qPCR of cultures grown under standardized laboratory conditions (n = 3 biological replicates per strain), normalized to 16S rRNA. For **c** and **d**, data are displayed as heatmaps of mean values across biological replicates. rRNA, ribosomal RNA.

To investigate the genomic differences underlying ear swelling responses, we genotyped *S. aureus* strains using a microarray-based kit targeting 336 genes, including agr alleles, resistance genes, and virulence factors (Figures 7 and 8). Next, we examined whether the presence of virulence factor genes correlated with ear swelling responses by comparing shared genes between strains that elicited stronger swelling and those that triggered milder reactions (Figure 9a). Our analysis revealed that AD2 and AD3, which induced lower immune responses, were highly similar genetically (96% similarity). In contrast, *S. aureus* strains AD1 and AD4—both of which triggered the strongest ear swelling—exhibited greater genomic variability. Specifically, AD1 shared 84–87% similarity with AD2 and AD3, whereas AD4 showed even lower similarity (74–77%). To further assess genetic relatedness, we performed *spa* typing on all strains. Consistent with the microarray results, AD2 and AD3 shared the same *spa* type (t084), AD1 and AD4 had distinct types (t1509 and t1451, respectively), and the reference strains Mu3 and Mu50 both belonged to *spa* type t002 (Table 2).

**Table 2:** *spa* Typing Results for Clinical Isolates and Reference Strains

Strain	*spa* Type (Protein A Gene)
Mu3	t002
Mu50	t002
AD1	t1509
AD2	t084
AD3	t084
AD4	t1451

The *spa* type corresponds to the sequence-based classification derived from the polymorphic X region of the *spa* gene, as determined by standard *spa* typing protocols. Identical *spa* types indicate strains with the same repeat pattern.

Notably, AD1 and AD4 harbored the agr quorum-sensing system I, whereas the other clinical strains carried agr system II (Figure 8a). However, the total number of virulence genes present in a strain did not directly correlate with ear swelling responses (Figure 7), suggesting that the mere presence of genes is insufficient to explain differences in inflammation.

Next, we next examined the virulence potential of the *S. aureus* strains by assessing the presence of key virulence genes (Geoghegan et al, 2018) and evaluating their transcriptional activity under both in vitro and in vivo conditions (Figure 9b–d). Microarray-based genotyping confirmed that all strains possessed a broad repertoire of virulence-associated genes, including spa (protein A), geh (glycerol ester hydrolase), aur (aureolysin), sspA/sspB (V8 and serine proteases), psmα (phenol-soluble modulins), isdA (iron-regulated surface determinant A), hla (α-hemolysin), and fnbpA (fibronectin-binding protein A). However, the presence of individual genes varied across isolates (Figure 9b).

Analysis of in vivo mRNA levels in ear tissue from colonized mice revealed substantial differences in virulence gene expression between strains (Figure 9c). Strains AD1 and AD4 exhibited the highest transcript levels of isdA, spa, sspB, psmα, and V8 protease. This indicates the active production of immune-modulatory and tissue-degrading factors during colonization. In contrast, AD2 displayed markedly lower in vivo expression of these genes, consistent with its reduced inflammatory phenotype. Notably, geh expression was significantly higher in AD1, Mu3, and Mu50 than in other strains. Despite carrying an extensive repertoire of virulence genes (Figure 9b) and showing moderate in vitro expression (Figure 9d), Mu3 and Mu50 exhibited comparatively lower in vivo expression of spa and protease genes, suggesting that host environmental factors may suppress their virulence activity.

Interestingly, although in vitro expression patterns were similar for some genes, the majority of virulence factors did not show a direct correlation between in vitro and in vivo expression. This indicates that broth culture–based in vitro assays may not accurately reflect the regulation and expression of virulence determinants during host colonization.

Collectively, these findings demonstrate that *S. aureus* strains differ in both their virulence gene content and their ability to activate key effectors within the host transcriptionally. The elevated in vivo expression of isdA, spa, geh, V8 protease, and sspB in AD1 and AD4 aligns with their increased inflammatory potential, which highlights the critical role of strain-specific regulatory mechanisms in shaping pathogenic outcomes.

### Synergistic modeling with MC903 and a clinical *S. aureus* strain exacerbates inflammation and recapitulates AD-like histological and immunological features

**Figure 10:**
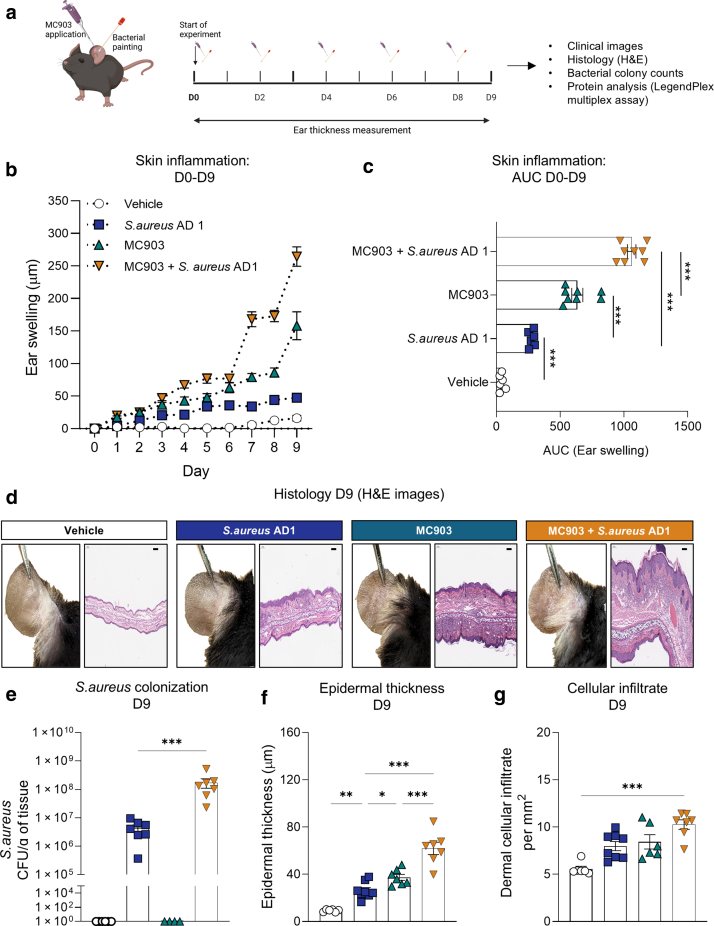
**Histological and inflammatory assessment after cotreatment with *S. aureus* AD1 and MC903.** (**a**) Experiment protocol. Mouse ear skin was topically inoculated with *S. aureus* strain AD1 after application of MC903 on days 0, 2, 4, 6, and 8. (**b**) Ear swelling measurements (μm) from D0 to D9, shown as mean values (n = 6–8 per group). (**c**) Cumulative ear swelling response represented as AUC from D0 to D9; individual values and group mean ± SEM are shown. Statistical significance was assessed using 1-way ANOVA followed by Tukey’s multiple comparisons test. (**d**) Representative clinical images (left) and H&E-stained histological sections (right) of mouse ear skin collected on D9. (**e**) Bacterial load of *S. aureus* recovered on D9, expressed as CFUs per gram of ear tissue. Statistical significance was assessed using Mann–Whitney test; ∗∗∗*P* < .001. (**f**) Quantification of epidermal thickness based on H&E-stained sections. Statistical significance was assessed using 1-way ANOVA followed by Tukey’s multiple comparisons test. ∗*P* < .05, ∗∗*P* < .01, and ∗∗∗*P* < .001. (**g**) Quantification of dermal cellular infiltration from the same histological sections. Statistical significance was assessed using the Kruskal–Wallis test followed by Dunn’s multiple comparisons test. ∗*P* < .05, ∗∗*P* < .01, and ∗∗∗*P* < .001. AUC, area under the curve; CFU, colony-forming unit; D0, day 0; D1, day 1; D2, day 2; D3, day 3; D4, day 4; D5, day 5; D6, day 6.

Our results, including histological analysis of mouse ear skin treated with *S. aureus* strains and virulence factor expression analysis, suggested that the *S. aureus* AD1 strain was an ideal candidate for developing an AD-like mouse model. To mimic the type 2 inflammation characteristic of AD, we utilized MC903 (calcipotriol), a vitamin D agonist widely used to induce AD-like skin lesions in mouse models. In our protocol, MC903 was applied first, followed by *S. aureus* AD1 treatment (30 minutes later), administered every alternate day over a 10-day period (Figure 10a).

**Figure 11:**
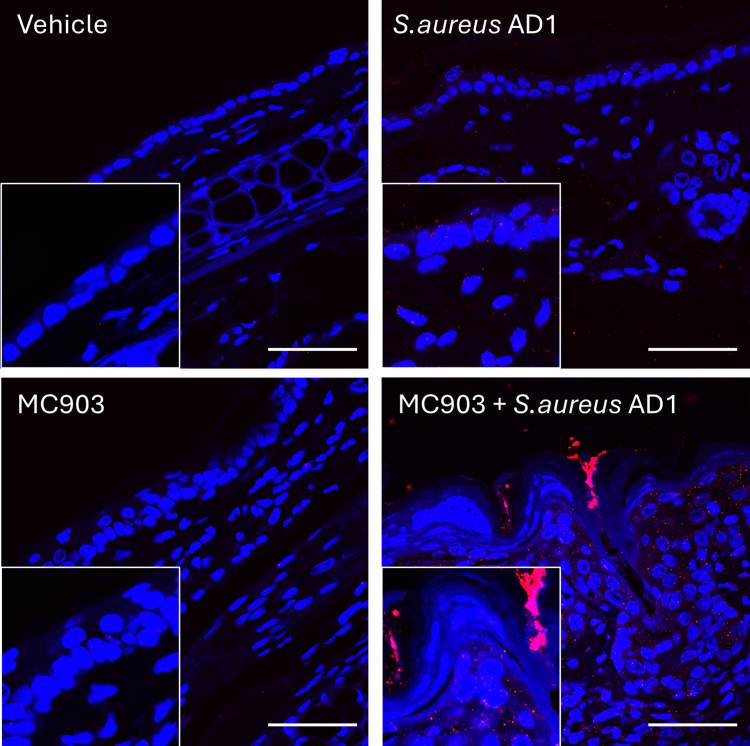
**Skin colonization by *S. aureus* in MC903-induced dermatitis.** Representative confocal images of skin sections from mice treated with vehicle, *S. aureus* AD1 alone, MC903 alone, or MC903 in combination with *S. aureus* AD1. Skin was collected on D9 and stained to visualize cell nuclei (DAPI, blue) and *S. aureus* (red). The insets show enlarged views of the epidermal regions in each condition. Bar = 50 μm. D9, day 9.

As early as day 4, mice treated with the combined *S. aureus* AD1 and MC903 (referred to as the mixed model) exhibited significantly higher ear swelling than mice treated with MC903 alone (*P* = .005) (Figure 10b). By day 9, the mixed model demonstrated the highest ear swelling levels overall, surpassing all other groups, including MC903 alone, *S. aureus* AD1 alone, and vehicle-treated controls, in that order (Figure 10b and c). Importantly, *S. aureus* colonization at day 9 was markedly elevated in the mixed model, with 10-fold higher bacterial loads than *S. aureus* AD1 alone (Figure 10e). This was also confirmed by *S. aureus*–specific immunostaining, which revealed greater bacterial abundance in both the epidermis and dermis of skin cotreated with MC903 and *S. aureus* (Figure 11).

**Figure 12:**
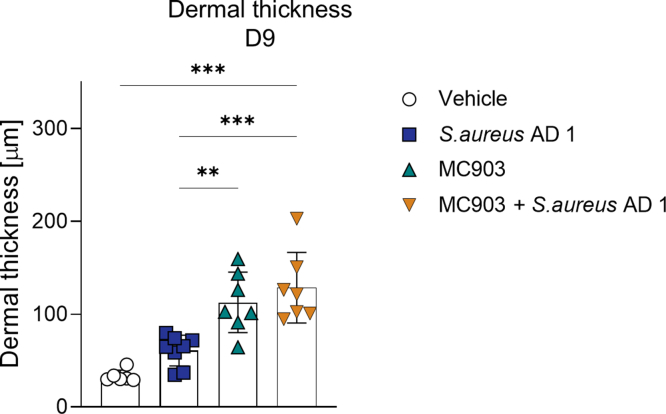
**Dermal skin thickness on day 9.** Dermal thickness was quantified from H&E-stained ear sections in 4 groups: vehicle, *S. aureus* AD1, MC903, and MC903 + *S. aureus* AD1. Bars show mean ± SEM, with individual values overlaid (n = 6–8 per group). Group differences were evaluated by 1-way ANOVA followed by Tukey’s posthoc test. ∗*P* < .05, ∗∗*P* < .01, and ∗∗∗*P* < .001. D9, day 9.

Histological analysis with H&E staining revealed increased epidermal thickness and cellular infiltration in *S. aureus* AD1–, MC903-, and mixed model–treated mice compared with that in vehicle-treated controls (Figures 10d–g and 12). In addition, the epidermal hyperplasia in the mixed model was most pronounced and featured mild fibrosis with vertical streaking of collagen in the papillary dermis, resembling the histopathological features observed in chronic AD (Schuler et al, 2024). These findings indicate that the mixed model not only enhances inflammation and *S. aureus* colonization but also replicates histological features resembling AD-like pathology.

**Figure 13:**
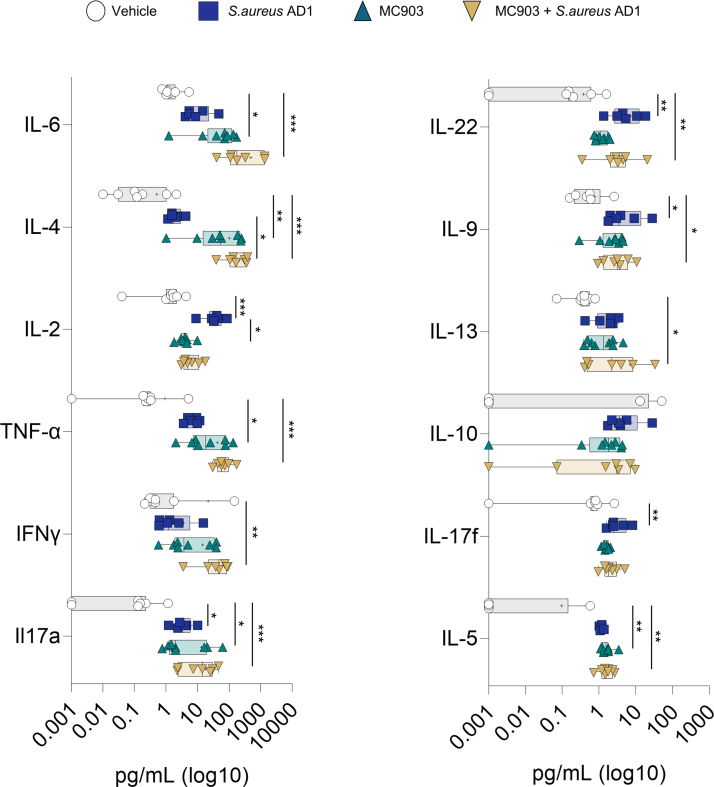
**Comparison of T helper cytokine levels in mouse ear skin under different treatment conditions.** Cytokine concentrations were quantified using the LEGENDplex Mouse T Helper Cytokine Panel. Data represent individual cytokine levels (pg/ml, log scale), normalized to the volume of skin tissue. Cytokines associated with Th1 (eg, IFN-γ, TNF-α), Th2 (eg, IL-4, IL-5, IL-13), Th17 (eg, IL-17A, IL-17F, IL-22), and regulatory or inflammatory responses (eg, IL-2, IL-6, IL-9, IL-10) are shown. Statistical significance was assessed using 1-way ANOVA followed by Tukey’s multiple comparisons test. ∗*P* < .05, ∗∗*P* < .01, and ∗∗∗*P* < .001. Th, T helper.

To further characterize the inflammatory responses, we analyzed protein levels of cytokines associated with type 1, type 2, type 22, and type 17 immune pathways across the treatment groups (Figure 13). IL-4, IL-5, and IL-13, hallmark cytokines of type 2 inflammation, were significantly elevated in both the MC903-treated and mixed model groups compared with those in the vehicle group. IL-6 and TNF-α exhibited the most pronounced increases in the mixed model compared with those in all other groups.

The type 1–associated cytokine IFN-γ was significantly higher in the mixed model than in the model with both vehicle and MC903 alone. Notably, IL-2 increased significantly in the *S. aureus* AD1–only group. Type 17 cytokines, including IL-17A, IL-17F, and IL-22, were elevated in both the *S. aureus* AD1 and mixed model groups but remained low in the MC903-alone group. This indicates a response that is more specifically associated with *S. aureus* exposure. IL-9 levels also increased in the mixed model compared with that in MC903 alone, contributing to the broader cytokine profile observed with cotreatment.

Taken together, these findings suggest that the combination of MC903 and *S. aureus* AD1 elicits a broader and more complex inflammatory response than MC903 alone, engaging multiple immune pathways. These results highlight the potential of *S. aureus* in shaping the inflammatory landscape of atopic-like skin conditions.

## Discussion

We investigated the inflammatory potential of *S. aureus* and *S. epidermidis* isolates obtained from AD lesions by applying it to mice with intact skin. We hypothesized that the combination of clinical *S. aureus* exposure with MC903-induced inflammation would more effectively mimic an AD-like phenotype and thus provide a more representative model than conventional AD models, which often do not involve *S. aureus*. Therefore, we isolated *S. aureus* and *S. epidermidis* strains from the lesions of patients with AD with varying degrees of disease severity (mild, moderate, and severe). We also included *S. aureus* strains Mu3 (ATCC 700698) and Mu50 (ATCC 700699), which are genetically similar to strain AD04.E17, which has recently been shown to induce an AD-like immunologic response (Braun et al, 2024; Byrd et al, 2017).

*S. epidermidis* is a common skin commensal that frequently colonizes the skin of patients with AD. Recent studies have shown that *S. epidermidis* can compromise the skin barrier in AD by secreting EcpA protease (Cau et al, 2021). However, emerging evidence suggests that not all *S. epidermidis* strains exhibit the same behaviour (Brown and Horswill, 2020; Linehan et al, 2018), highlighting the need to explore strain-specific differences in AD.

Our results show that clinical *S. epidermidis* strains from AD lesions can induce inflammatory responses in mice with intact skin. However, these responses are much less pronounced than the stronger inflammation induced by *S. aureus* strains isolated from the same lesional sites (Figures 1 and 3). Furthermore, strain-specific variations in inflammation were absent in *S. epidermidis* (Figures 1c and 3a and b). This underscores the differential role of *S. epidermidis* in AD, possibly acting as a cofactor for disease progression rather than a primary trigger. The *S. epidermidis* genome is composed of approximately 80% core genes and 20% variable genes (Méric et al, 2015). These variable genes are acquired through horizontal gene transfer facilitated by mobile genetic elements such as phages and plasmids. Notably, the likelihood of horizontal gene transfer among *S. epidermidis* populations on the skin is considerable, highlighting the dynamic genetic adaptability of this bacteria (Zhou et al, 2020). In addition, the virulence of *S. aureus* strains has been shown to be influenced by the quorum-sensing system of *S. epidermidis* in pediatric patients with AD (Williams et al, 2019; Zhou et al, 2023). This suggests that the presence of specific *S. epidermidis* strains alongside *S. aureus* may modify or enhance inflammatory potential. This hypothesis merits further investigation in future studies.

The AD-like immunological effects of clinical *S. aureus* strains have been shown to vary depending on the specific strain isolated but do not strictly correlate with the disease severity of the source patient (Byrd et al, 2017). Furthermore, it is suggested that these differences may also be influenced by host factors such as sequence variation in the barrier genes (eg, FLG), sex, age, race, immune profile, etc (Byrd et al, 2017; Cho et al, 2001; Rauer et al, 2023). Our results support this notion because we observed different inflammatory responses induced by *S. aureus* strains (Figure 1b and c). In addition, these variations were not strictly related to the severity of the patient with AD from whom the strains were isolated (Table 1). This suggests that host-specific factors, in addition to strain-specific characteristics, may play a critical role in shaping the differential inflammatory responses to *S. aureus* strains. We also observed minimal inflammation in the vehicle-treated group. This inflammation was primarily attributable to the handling procedure rather than the vehicle itself (Figure 4). It was also accompanied by alteration in the local skin microbiota. These findings underscore the importance of considering handling-related effects when interpreting baseline skin responses.

The genomic profiles of *S. aureus* strains differ between those isolated from AD lesions and those isolated from healthy skin (Wang et al., 2024b). Notably, *S. aureus* strains from AD are enriched with virulence genes (Saheb Kashaf et al, 2023). However, there is limited understanding of how interstrain genomic variability among *S. aureus* strains in AD relates to their pathogenic potential. In our genotyping analysis on a limited panel of clinical isolates, we observed differences in certain virulence-associated genes between strains with stronger inflammatory responses and those with milder effects (Figures 8 and 9b). Examining categories of virulence factors, including proteases, enterotoxins, and adhesion factors, revealed differences in the number of genes within these categories (Figure 7). However, these variations did not consistently correlate with inflammatory severity. Instead, our findings support the growing body of evidence suggesting that the expression of specific virulence factors may be more predictive of inflammatory potential than the mere presence of genes (Figure 9c) (Braun et al, 2024). In addition, our results indicate that in vivo virulence factor expression differs from that observed in in vitro broth cultures, providing a more accurate reflection of the strains’ virulence potential (Figure 9c and d). Further validation using mutant strains would be essential to clarify the role of specific virulence genes such as geh, isdA, V8 protease, and psmα in driving inflammation. Although our targeted microarray efficiently screened known genes, its limited scope may overlook novel or divergent variants. Whole-genome sequencing could provide broader insight in future studies.

The longitudinal application of MC903 is currently a widely used method to induce AD-like disease in mouse models (Gilhar et al, 2021; Li et al, 2006; Moosbrugger-Martinz et al, 2017). Short-term application of MC903 is sufficient to prime the skin for a subclinical type 2 immune response by inducing thymic stromal lymphopoietin (Li et al, 2006; Naidoo et al, 2018). Introducing a clinical *S. aureus* strain into this preconditioned environment, characterized by an inherent predisposition, may provide a more accurate model of events in human AD. Our AD-like model, generated by coapplication of MC903 and a clinical *S. aureus* strain, resulted in a 10-fold increase in bacterial colonization compared with *S. aureus* alone (Figure 10e). This aligns with the known ability of *S. aureus* to exploit barrier dysfunction in atopic skin (Nakatsuji et al, 2016). Importantly, we also observed bacterial penetration into the dermis, a feature that closely mirrors disease pathology in human AD (Figure 11). This pronounced increase in colonization and tissue invasion underscores the model’s value as a robust platform for investigating host–microbe interactions and for evaluating targeted anti–*S. aureus* therapies.

Multiple immune pathways contribute to the pathogenesis of AD, including type 1, type 2, type 17, and type 22 responses, with type 2 immunity being dominant in most endotypes (Czarnowicki et al, 2019). Previous studies have investigated how *S. aureus* influences type 2 inflammation in MC903-induced AD models. However, no study has specifically explored the inflammatory response triggered by a clinical inflammatory strain of *S. aureus* introduced during subclinical type 2 inflammation or systematically profiled cytokine signatures under such conditions. Using our model with clinical *S. aureus* (AD1 strain) and MC903, we observed an immune response that was primarily skewed toward a type 2 cytokine profile, with additional contributions from type 1, type 17, and type 22 cytokines (Figure 13). Several of these cytokine markers were negligible or less pronounced when MC903 or *S. aureus* AD1 was applied alone. Type 2 cytokine levels were minimal in the *S. aureus* AD1–only model, whereas type 17 and type 22 responses remained low in the MC903-alone condition. Moreover, cytokine expression in these individual models was substantially lower than in the combined treatment group, where coexposure led to a more robust and diverse inflammatory signature. A potential factor contributing to the heightened immune responses in this model might be the modulation of IL-2 expression, which is known to influence both pro and anti-inflammatory pathways. We observed that IL-2 levels were highest in the presence of *S. aureus* AD1 but lower in the mouse model with MC903 and *S. aureus* AD1. IL-2 has a dual role: (i) maintaining regulatoy T cells to regulate immune responses and (ii) stimulating conventional T cells to promote immune activation. IL-2 has also been explored as a potential therapeutic immunosuppressive molecule (Abbas et al, 2018). Interestingly, mast cell–derived IL-2 has been shown to attenuate allergic dermatitis (Hershko et al, 2011), and therapy using IL-2 or its agonist has demonstrated a reduction in clinical symptoms of AD (Hsieh et al, 1991; Silverberg et al, 2024).

It is important to note that the protein-based LEGENDplex cytokine assay used in this study does not allow for the direct identification of the cellular source of each cytokine. Although the data provide valuable insight into pathway-level activation, further studies are necessary to define the specific immune cell types responsible for these responses. These studies could include single-cell analyses or flow cytometry. In addition, expanding the in vivo model to include a wider range of clinical *S. aureus* strains would provide a broader assessment of inflammatory potential and enhance the model’s translational relevance.

Overall, our findings indicate a clear synergistic effect in our refined AD model, where the combined application of MC903 and clinical *S. aureus* strain (AD1) triggers a more comprehensive immune response resembling human AD.

### Limitations

This study has some limitations that should be considered. Whereas *S. aureus* strains in AD lesions are typically clonal, *S. epidermidis* strains are genetically diverse (Byrd et al, 2017). However, we isolated only a single *S. epidermidis* clone per site. This approach was chosen owing to practical constraints, such as sampling feasibility, but it may not fully capture the potential variability in immune interactions among different clones. Future studies should explore whether multiple clones from the same site exhibit varying immunomodulatory effects. In addition, our mRNA expression analysis focused on a subset of virulence factors, meaning that other relevant contributors, such as toxin production, metabolic activity, interspecies interactions, and host immune responses, were not assessed. Because these factors may also influence inflammation, future research should incorporate a broader range of virulence determinants to provide a more comprehensive understanding of bacterial contributions to AD. Despite these limitations, our findings provide valuable functional insights into strain-specific differences and lay the foundation for further research on their role in disease progression and potential therapeutic strategies.

In conclusion, our findings highlight the crucial role of strain variability in *S. aureus–*induced skin inflammation. By characterizing these effects in intact mouse skin and together with topical application of MC903, we have established a mouse model that recapitulates key immune responses involving type 2, type 17, type 22, and type 1 cytokines, along with clinical and histological features of AD. This model can serve as a valuable tool to further investigate *S. aureus–*mediated mechanisms in AD and explore microbiome-targeted therapeutic strategies for AD.

## Materials and Methods

### Bacterial isolates

Clinical bacterial isolates were isolated by swabbing lesional sites of patients with AD using ESwab 482C (Copan Diagnostics) and storing the samples in Amies medium for a few hours before processing. A 200 μl aliquot of the Amies liquid medium was plated onto 5% sheep blood agar and Chocolate PVX agar (BioMérieux) and incubated at 37 °C for 18 hours. Bacterial colonies were identified using matrix-assisted laser desorption ionization–time of flight mass spectrometry, with *S. aureus* further confirmed on chromID *S. aureus* Elite agar (BioMérieux). After identification, bacterial isolates were stored in CRYOBANK (Mast Group) and maintained in a −80 °C freezer until further use. Two nonclinical reference strains—*S. aureus* Mu3 (ATCC 700698) and *S. aureus* Mu50 (ATCC 700699)—were included in the study. Clinical bacterial isolates were obtained from patients undergoing a clinical study that was approved by the Ethics Committee of the Medical University of Graz (EK number: 27-263 ex 14/15, 1092-2015) and the study protocol UV-Mikrobiom_2015, version 3.0.

### Bacterial cultures

The bacterial cultures were grown in TSB (catalog number 1.05459, Millipore Sigma) for 16 hours at 37 °C with shaking at 200 r.p.m. The optical density of the cultures was measured and adjusted to 1.0 with TSB. Bacterial concentrations were determined using the serial dilution method, in which a series of 10-fold dilutions were prepared, aliquots were plated on Tryptic Soy Agar (catalog number 1.05458, Millipore Sigma), and the plates were incubated for 24 hours at 37 °C under aerobic conditions. Aliquots of 1 ml bacterial suspension were prepared and stored at −80 °C for later use.

### Bacteria and MC903–induced skin inflammation

Ethical approval for all animal experiments was granted by the Austrian Federal Ministry of Education, Science and Research (approval number GZ BMBWF-66.010/0147-V/3b/2018). All procedures were conducted under specific pathogen-free and biosafety level 2 (S2) conditions. To induce skin inflammation, bacteria were thawed and painted topically to both sides of the mouse ears. For experiments involving MC903, 1 nM compound was applied to both sides of the ears 30 minutes before introducing the *S. aureus* AD1. The bacterial suspension was applied using a swab, which was gently rubbed across the ear surfaces until they appeared evenly moistened. This procedure was carried out multiple times on the designated days. Inflammatory responses were evaluated by measuring ear thickness with a micrometer (Mitutoyo). The extent of ear swelling was determined using the following formula: ear swelling = thickness on day *x* − thickness on day 0.

### Bacterial abundance

Mouse ear skin tissue samples were placed into MagNA Lyser tubes prefilled with ceramic beads (catalog number 03358941001, Roche Life Sciences) containing 1 ml of sterile PBS. Mechanical lysis was performed using a MagNA Lyser instrument at 6000 r.p.m. for 30 seconds to ensure thorough tissue disruption and bacterial release. The resulting lysate was serially diluted in PBS and plated onto either chromID *S. aureus* Elite agar (BioMérieux) for selective detection of *S. aureus* or mannitol salt agar plates for detection of *S. epidermidis* (catalog number M9052, Sigma-Aldrich). Plates were incubated overnight at 37 °C to allow bacterial colony formation. Bacterial abundance was quantified by counting visible colonies using ImageJ software, and results were expressed as CFU/g of tissue.

### *S. aureus* genotyping

Genotyping of all isolates was conducted using the microarray-based Genotyping Kit - *S. aureus* according to the manual version 1012012100004 (INTER-ARRAY fzmb GmbH). *S. aureus* was cultured overnight at 37 °C on Columbia blood agar. One loop (1 mm in diameter) of staphylococcal cells (approximately 1–5 × 10^6^) was harvested and digested in a lysis reagent containing lysozyme, lysostaphin, and RNAse A. After this, a second lysis step and DNA purification were carried out using the DNeasy Blood & Tissue Kit (Qiagen), according to the manufacturer's instructions. Linear amplification was performed using 1 primer for each target sequence. This DNA microarray comprises 336 target sequences, which correspond to distinct genes and their allelic variants, including *S. aureus*–specific genes, accessory gene regulator (agr) alleles, resistance genes, and genes coding for virulence factors (toxins, enterotoxins, hemolysins, proteases, biofilm formation molecules). Data acquisition and analysis were done with the INTERVISION-Reader.

### qPCR protocol

Total RNA was extracted from mouse skin tissue and bacterial pellets (grown in TSB for 18 hours) using the miRNeasy Micro Kit (number 217084, Qiagen) and RNeasy Protect Bacteria Mini Kit (number 74524, Qiagen), respectively. RNA concentration and purity were assessed with a Nanodrop. cDNA was synthesized from 500 ng of RNA using the RevertAid First Strand cDNA Synthesis Kit (K1621, Thermo Fisher Scientific) with random hexamer primers, following the manufacturer’s instructions. qPCR was performed using Bio-Rad CFX96 and GoTag qPCR Master Mix (A6001, Promega). Primer sequences are listed in Table 3 (Poh et al., 2022). Relative gene expression levels were quantified as mRNA levels normalized to 16S ribosomal RNA expression.

**Table 3:** Primers Used for qpcr Analysis in this Study

Number	Gene	Target	Sequence	Sequence for qPCR
1	16s	Pan bacterial	Forward	CGGTGAATACGTTCCCGG
			Reverse	TACGGCTACCTTGTTACGACTT
2	16s	*Staphylococcus* specific	Forward	TGAGTGATGAAGGTCTTCGGATC
			Reverse	ATAACGCTTGCCACCTACGTATTAC
3	sspB	Staphopain B	Forward	TGCGCTAGCAGTTGTTGGTA
			Reverse	ACCTATCATTGAACCATACCAGT
4	V8	V8 (sspA) protease	Forward	ACCTGTAGCAACAATGTGGGA
			Reverse	ATTTGGTACACCGCCCCAAT
5	splA	SplA serine protease	Forward	TGGTAGCATTTGTGGGTGGT
			Reverse	GTCGTAGTTTCCTCCGCCTT
6	aur	Aureolysin	Forward	TGCAACCGAGTGTTGATGGT
			Reverse	TAAATGCTTTGTCGGCTGCG
7	geh	Lipase	Forward	GCAACCACGGACAAACATCC
			Reverse	TCTGTCGGTTTCTCTGGGGA
8	hysA	Hyaluronate lyase	Forward	TGAGGAACACATGGCTGGAC
			Reverse	TTTCCGCTCCTGACCACAAG
9	hla	Alpha hemolysin	Forward	TGGTTTAGCCTGGCCTTCAG
			Reverse	ATTTGCACCAATAAGGCCGC
10	pmsa	Phenol soluble modulin A	Forward	TAAGCTTAATCGAACAATTC
			Reverse	CCCCTTCAAATAAGATGTTCATATC
11	isdA	Iron-regulated surface determinant (Isd)	Forward	CCAACAAGTCAATGCGGCAA
			Reverse	TGTGTGACTTCTCTGAAGAGCC
12	spa	Protein A	Forward	GCGACGACGTCCAGCTAATA
			Reverse	AAGCAACCAGCAAACCATGC
13	fnbpA	Fibronectin binding protein A	Forward	AGTTTCTGGCTCACTCGGC
			Reverse	AGCGGCCAAAATGAAGGTCA

The table lists the forward and reverse primer sequences used for RT-qPCR targeting selected genes of *S. aureus*.

### Histological analysis

Quantification of epidermal thickness, dermal thickness, and cellular infiltrate was conducted using QuPath (version 0.5.1) on H&E-stained tissue sections. Epidermal and dermal regions of interest were manually delineated in QuPath, and thickness was measured at regular intervals using the software’s measurement tool, with mean values calculated for each region. Cellular infiltrate density was assessed within dermal regions of interest using QuPath’s cell detection tool, optimized for hematoxylin-stained nuclei, and expressed as cells per mm^2^.

### Protein

Cytokine levels were measured using the LEGENDplex Mouse T Helper Cytokine Panel version 3 (number 741044, BioLegend), following the manufacturer's instructions. Protein isolation was carried out using the T-PER Tissue Protein Extraction Reagent (78510, Thermo Fisher Scientific) supplemented with Protease Inhibitor (A32965, Thermo Fisher Scientific). The protein lysates were normalized by volume to ensure consistency and prepared for downstream analysis.

Protein lysates were loaded onto LEGENDplex plates according to the instructions provided by the manufacturer. Samples were analyzed using flow cytometer (CytoFLEX S, Beckman Coulter). Cytokine concentrations were quantified by comparing the sample readings with a standard curve and using the LEGENDplex software (www.biolegend.com/en-us/legendplex).

### Statistical analysis

All analyses were performed using GraphPad Prism, version 9.5.1 (GraphPad Software, San Diego, California, https://www.graphpad.com/). Data normality was assessed using the Shapiro–Wilk test (α = 0.05). For normally distributed datasets, 1-way ANOVA followed by Tukey’s posthoc test was used to assess group differences. For non-normally distributed data, the Kruskal–Wallis test with Dunn’s posthoc correction was applied. Differences in *S. aureus* colonization levels (CFU/ml) and ear thickness on day 4 between groups were evaluated using unpaired *t*-tests. Data are presented as mean ± SEM, except for cytokine measurements, which are shown as mean with minimum–maximum range.

## Ethics Statement

Clinical bacterial isolates were obtained from participants enrolled in a study approved by the Ethics Committee of the Medical University of Graz (EK number: 27-263 ex 14/15, 1092-2015), in accordance with the study protocol UV-Mikrobiom_2015, version 3.0. All donors provided written, informed consent prior to participation. All animal experiments were conducted with prior approval from the Austrian Federal Ministry of Education, Science and Research (approval number GZ BMBWF-66.010/0147-V/3b/2018).

## Data Availability Statement

The bacterial strains used in this study, except for Mu3 and Mu50 (which are commercially available), will be made available upon request, provided that the requesting party covers the transportation costs and supplies the necessary import documentation. The data that support the findings of this study are available from the corresponding and the first author (vijaykumar.patra@gmail.com/aaroh.joshi@medunigraz.at) upon request.

## ORCIDs

Aaroh Joshi: http://orcid.org/0000-0003-4385-1318

Altan Cornu: http://orcid.org/0009-0006-6715-2246

Gernot Zarfel: http://orcid.org/0000-0003-1592-4559

Camille Braun: http://orcid.org/0000-0001-9260-8977

Jean-Francois Nicolas: http://orcid.org/0000-0003-4204-803X

Richard L. Gallo: http://orcid.org/0000-0002-1401-7861

Marc Vocanson: http://orcid.org/0000-0002-0181-8862

Peter Wolf: http://orcid.org/0000-0001-7777-9444

Vijaykumar Patra: http://orcid.org/0000-0002-7161-7767

## Conflict of Interest

The authors have no proprietary or commercial interest in any materials discussed in this article. RLG is a cofounder, scientific advisor, consultant, and equity holder of MatriSys Biosciences. The remaining authors state no conflcit of interest.

## References

[bib1] Abbas A.K., Trotta E., R Simeonov D., Marson A., Bluestone J.A. (2018). Revisiting IL-2: biology and therapeutic prospects. Sci Immunol.

[bib2] Beck L.A., Cork M.J., Amagai M., De Benedetto A., Kabashima K., Hamilton J.D. (2022). Type 2 inflammation contributes to skin barrier dysfunction in atopic dermatitis. JID Innov.

[bib3] Braun C., Badiou C., Guironnet-Paquet A., Iwata M., Lenief V., Mosnier A. (2024). Staphylococcus aureus-specific skin resident memory T cells protect against bacteria colonization but exacerbate atopic dermatitis-like flares in mice. J Allergy Clin Immunol.

[bib4] Brown M.M., Horswill A.R. (2020). Staphylococcus epidermidis-Skin friend or foe?. PLoS Pathog.

[bib5] Brunner P.M., Guttman-Yassky E. (2019). Racial differences in atopic dermatitis. Ann Allergy Asthma Immunol.

[bib6] Byrd A.L., Deming C., Cassidy S.K.B., Harrison O.J., Ng W.I., Conlan S. (2017). Staphylococcus aureus and Staphylococcus epidermidis strain diversity underlying pediatric atopic dermatitis. Sci Transl Med.

[bib7] Cau L., Williams M.R., Butcher A.M., Nakatsuji T., Kavanaugh J.S., Cheng J.Y. (2021). Staphylococcus epidermidis protease EcpA can be a deleterious component of the skin microbiome in atopic dermatitis. J Allergy Clin Immunol.

[bib8] Cho S.H., Strickland I., Tomkinson A., Fehringer A.P., Gelfand E.W., Leung D.Y. (2001). Preferential binding of Staphylococcus aureus to skin sites of Th2-mediated inflammation in a murine model. J Invest Dermatol.

[bib9] Czarnowicki T., He H., Krueger J.G., Guttman-Yassky E. (2019). Atopic dermatitis endotypes and implications for targeted therapeutics. J Allergy Clin Immunol.

[bib10] Deckers I.A., McLean S., Linssen S., Mommers M., van Schayck C.P., Sheikh A. (2012). Investigating international time trends in the incidence and prevalence of atopic eczema 1990-2010: a systematic review of epidemiological studies. PLoS One.

[bib11] Eichenseher F., Herpers B.L., Badoux P., Leyva-Castillo J.M., Geha R.S., van der Zwart M. (2022). Linker-improved chimeric endolysin selectively kills Staphylococcus aureus in vitro, on reconstituted human epidermis, and in a murine model of skin infection. Antimicrob Agents Chemother.

[bib12] Fallon P.G., Sasaki T., Sandilands A., Campbell L.E., Saunders S.P., Mangan N.E. (2009). A homozygous frameshift mutation in the mouse Flg gene facilitates enhanced percutaneous allergen priming. Nat Genet.

[bib13] Geoghegan J.A., Irvine A.D., Foster T.J. (2018). Staphylococcus aureus and atopic dermatitis: a complex and evolving relationship. Trends Microbiol.

[bib14] Gilhar A., Reich K., Keren A., Kabashima K., Steinhoff M., Paus R. (2021). Mouse models of atopic dermatitis: a critical reappraisal. Exp Dermatol.

[bib15] Hershko A.Y., Suzuki R., Charles N., Alvarez-Errico D., Sargent J.L., Laurence A. (2011). Mast cell interleukin-2 production contributes to suppression of chronic allergic dermatitis. Immunity.

[bib16] Hsieh K.H., Chou C.C., Huang S.F. (1991). Interleukin 2 therapy in severe atopic dermatitis. J Clin Immunol.

[bib17] Jin H., He R., Oyoshi M., Geha R.S. (2009). Animal models of atopic dermatitis. J Invest Dermatol.

[bib18] Joshi A.A., Vocanson M., Nicolas J.F., Wolf P., Patra V. (2023). Microbial derived antimicrobial peptides as potential therapeutics in atopic dermatitis. Front Immunol.

[bib19] Kennedy E.A., Connolly J., Hourihane J.O., Fallon P.G., McLean W.H.I., Murray D. (2017). Skin microbiome before development of atopic dermatitis: early colonization with commensal staphylococci at 2 months is associated with a lower risk of atopic dermatitis at 1 year. J Allergy Clin Immunol.

[bib20] Key F.M., Khadka V.D., Romo-González C., Blake K.J., Deng L., Lynn T.C. (2023). On-person adaptive evolution of Staphylococcus aureus during treatment for atopic dermatitis. Cell Host Microbe.

[bib21] Kim D., Kobayashi T., Nagao K. (2019). Research techniques made simple: mouse models of atopic dermatitis. J Invest Dermatol.

[bib22] Lai Y., Di Nardo A., Nakatsuji T., Leichtle A., Yang Y., Cogen A.L. (2009). Commensal bacteria regulate toll-like receptor 3-dependent inflammation after skin injury. Nat Med.

[bib23] Landemaine L., Da Costa G., Fissier E., Francis C., Morand S., Verbeke J. (2023). Staphylococcus epidermidis isolates from atopic or healthy skin have opposite effect on skin cells: potential implication of the AHR pathway modulation. Front Immunol.

[bib24] Lane Starr N.M., Al-Rayyan N., Smith J.M., Sandstrom S., Swaney M.H., Salamzade R. (2024). Combined metagenomic- and culture-based approaches to investigate bacterial strain-level associations with medication-controlled mild-moderate atopic dermatitis. J Allergy Clin Immunol Glob.

[bib25] Leyden J.J., Marples R.R., Kligman A.M. (1974). Staphylococcus aureus in the lesions of atopic dermatitis. Br J Dermatol.

[bib26] Leyva-Castillo J.M., McGurk A., Geha M.D.R. (2020). Allergic skin inflammation and S. aureus skin colonization are mutually reinforcing. Clin Immunol.

[bib27] Li M., Hener P., Zhang Z., Kato S., Metzger D., Chambon P. (2006). Topical vitamin D3 and low-calcemic analogs induce thymic stromal lymphopoietin in mouse keratinocytes and trigger an atopic dermatitis. Proc Natl Acad Sci USA.

[bib28] Linehan J.L., Harrison O.J., Han S.J., Byrd A.L., Vujkovic-Cvijin I., Villarino A.V. (2018). Non-classical immunity controls microbiota impact on skin immunity and tissue repair. Cell.

[bib29] Matsuda H., Watanabe N., Geba G.P., Sperl J., Tsudzuki M., Hiroi J. (1997). Development of atopic dermatitis-like skin lesion with IgE hyperproduction in NC/Nga mice. Int Immunol.

[bib30] Matsuoka H., Maki N., Yoshida S., Arai M., Wang J., Oikawa Y. (2003). A mouse model of the atopic eczema/dermatitis syndrome by repeated application of a crude extract of house-dust mite Dermatophagoides farinae. Allergy.

[bib31] Méric G., Miragaia M., de Been M., Yahara K., Pascoe B., Mageiros L. (2015). Ecological overlap and horizontal gene transfer in Staphylococcus aureus and Staphylococcus epidermidis. Genome Biol Evol.

[bib32] Meylan P., Lang C., Mermoud S., Johannsen A., Norrenberg S., Hohl D. (2017). Skin colonization by Staphylococcus aureus precedes the clinical diagnosis of atopic dermatitis in infancy. J Invest Dermatol.

[bib33] Moosbrugger-Martinz V., Schmuth M., Dubrac S. (2017). A Mouse Model for Atopic Dermatitis Using Topical Application of Vitamin D3 or of Its Analog MC903. Methods Mol Biol.

[bib34] Naidoo K., Jagot F., van den Elsen L., Pellefigues C., Jones A., Luo H. (2018). Eosinophils determine dermal thickening and water loss in an MC903 model of atopic dermatitis. J Invest Dermatol.

[bib35] Nakatsuji T., Chen T.H., Two A.M., Chun K.A., Narala S., Geha R.S. (2016). Staphylococcus aureus exploits epidermal barrier defects in atopic dermatitis to trigger cytokine expression. J Invest Dermatol.

[bib36] Nakatsuji T., Hata T.R., Tong Y., Cheng J.Y., Shafiq F., Butcher A.M. (2021). Development of a human skin commensal microbe for bacteriotherapy of atopic dermatitis and use in a phase 1 randomized clinical trial. Nat Med.

[bib37] Obata S., Hisatsune J., Kawasaki H., Fukushima-Nomura A., Ebihara T., Arai C. (2023). Comprehensive genomic characterization of Staphylococcus aureus isolated from atopic dermatitis patients in Japan: correlations with disease severity, eruption type, and anatomical site. Microbiol Spectr.

[bib38] Okamoto H., Li S., Nakamura Y. (2025). The role of skin dysbiosis and quorum sensing in atopic dermatitis. JID Innov.

[bib39] Paller A.S., Kong H.H., Seed P., Naik S., Scharschmidt T.C., Gallo R.L. (2019). The microbiome in patients with atopic dermatitis. J Allergy Clin Immunol.

[bib57] Poh S.E., Koh W.L.C., Lim S.Y.D., Wang E.C.E., Yew Y.W., Common J.E.A. (2022). Expression of *Staphylococcus aureus* virulence factors in atopic dermatitis. JID Innov.

[bib40] Rauer L., Reiger M., Bhattacharyya M., Brunner P.M., Krueger J.G., Guttman-Yassky E. (2023). Skin microbiome and its association with host cofactors in determining atopic dermatitis severity. J Eur Acad Dermatol Venereol.

[bib41] Saheb Kashaf S., Harkins C.P., Deming C., Joglekar P., Conlan S., Holmes C.J. (2023). Staphylococcal diversity in atopic dermatitis from an individual to a global scale. Cell Host Microbe.

[bib42] Scharschmidt T.C., Man M.Q., Hatano Y., Crumrine D., Gunathilake R., Sundberg J.P. (2009). Filaggrin deficiency confers a paracellular barrier abnormality that reduces inflammatory thresholds to irritants and haptens. J Allergy Clin Immunol.

[bib43] Schuler C.F., Tsoi L.C., Billi A.C., Harms P.W., Weidinger S., Gudjonsson J.E. (2024). Genetic and immunological pathogenesis of atopic dermatitis. J Invest Dermatol.

[bib44] Silverberg J.I., Rosmarin D., Chovatiya R., Bieber T., Schleicher S., Beck L. (2024). The regulatory T cell-selective interleukin-2 receptor agonist rezpegaldesleukin in the treatment of inflammatory skin diseases: two randomized, double-blind, placebo-controlled phase 1b trials. Nat Commun.

[bib45] Simpson E.L., Villarreal M., Jepson B., Rafaels N., David G., Hanifin J. (2018). Patients with atopic dermatitis colonized with Staphylococcus aureus Have a distinct phenotype and endotype. J Invest Dermatol.

[bib46] Spergel J.M., Mizoguchi E., Brewer J.P., Martin T.R., Bhan A.K., Geha R.S. (1998). Epicutaneous sensitization with protein antigen induces localized allergic dermatitis and hyperresponsiveness to methacholine after single exposure to aerosolized antigen in mice. J Clin Invest.

[bib47] Volz T., Kaesler S., Draing C., Hartung T., Röcken M., Skabytska Y. (2018). Induction of IL-10-balanced immune profiles following exposure to LTA from Staphylococcus epidermidis. Exp Dermatol.

[bib48] Wang Z., Hülpüsch C., Foesel B., Traidl-Hoffmann C., Reiger M., Schloter M. (2024). Genomic and functional divergence of Staphylococcus aureus strains from atopic dermatitis patients and healthy individuals: insights from global and local scales. Microbiol Spectr.

[bib49] Wang Z., Peng X., Hülpüsch C., Khan Mirzaei M., Reiger M., Traidl-Hoffmann C. (2024). Distinct prophage gene profiles of Staphylococcus aureus strains from atopic dermatitis patients and healthy individuals. Microbiol Spectr.

[bib50] Weidinger S., Beck L.A., Bieber T., Kabashima K., Irvine A.D. (2018). Atopic dermatitis. Nat Rev Dis Primers.

[bib51] Weiss A., Delavenne E., Matias C., Lagler H., Simon D., Li P. (2022). Topical niclosamide (ATx201) reduces Staphylococcus aureus colonization and increases Shannon diversity of the skin microbiome in atopic dermatitis patients in a randomized, double-blind, placebo-controlled Phase 2 trial. Clin Transl Med.

[bib52] Williams M.R., Costa S.K., Zaramela L.S., Khalil S., Todd D.A., Winter H.L. (2019). Quorum sensing between bacterial species on the skin protects against epidermal injury in atopic dermatitis. Sci Transl Med.

[bib53] Williams M.R., Gallo R.L. (2017). Evidence that human skin microbiome dysbiosis promotes atopic dermatitis. J Invest Dermatol.

[bib54] Yi Pan M.H., Bordag N., Absenger-Novak M., Strobl H., Bieber T., Wolf P. (2025). Live but not heat-killed Staphylococcus epidermidis elicit an anti-inflammatory phenotype in human embryonic Langerhans cells. SKINdeep.

[bib55] Zhou W., Spoto M., Hardy R., Guan C., Fleming E., Larson P.J. (2020). Host-specific evolutionary and transmission dynamics shape the functional diversification of Staphylococcus epidermidis in human skin. Cell.

[bib56] Zhou Y., Xu X., Liu Y., Wang A., Luo Y., Liu X. (2023). Heterogeneous regulation of staphylococcus aureus by different staphylococcus epidermidis agr types in atopic dermatitis. J Invest Dermatol.

